# Experimentally Validated Quantum-Secure Federated Learning over a Multi-user Quantum Network

**DOI:** 10.34133/research.1299

**Published:** 2026-06-10

**Authors:** Zhi-Ping Liu, Xiao-Yu Cao, Hao-Wen Liu, Xiao-Ran Sun, Yu Bao, Jian-Yu Shen, Yu-Shuo Lu, Hua-Lei Yin, Zeng-Bing Chen

**Affiliations:** ^1^ National Laboratory of Solid State Microstructures and School of Physics, Collaborative Innovation Center of Advanced Microstructures, Nanjing University, Nanjing 210093, China.; ^2^ School of Physics and Key Laboratory of Quantum State Construction and Manipulation (Ministry of Education), Renmin University of China, Beijing 100872, China.

## Abstract

Federated learning enables decentralized, privacy-preserving training but remains vulnerable to privacy leakage in the quantum era. Quantum federated learning (QFL) offers a promising path toward enhanced security and efficiency. However, a practical and experimentally validated QFL protocol utilizing near-term quantum techniques to address data privacy has been lacking. Here, we present QuNetQFL, a QFL protocol implemented on quantum networks, in which local model updates are masked with distributed quantum secret keys, offering information-theoretic security during aggregation. We experimentally validate the protocol on a 4-client quantum network and benchmark its performance using the generated keys on quantum and real-world datasets. Adding a single quantum client substantially improves global accuracy for classifying multipartite entangled and nonstabilizer quantum datasets. For language tasks, we apply QuNetQFL to sentiment analysis by federated fine-tuning of a hybrid classical-quantum language model, achieving comparable and robust performance in simulation and on real quantum hardware. Large-scale simulations further demonstrate scalability to 200 clients for handwritten-digit recognition, with rapid convergence and a 75% reduction in communication cost via model compression. Our work establishes a practical and scalable route to quantum-secure federated learning for the emerging quantum internet.

## Introduction

Deep learning has achieved remarkable success in fields [1] such as disease diagnosis [2], autonomous driving [3], and tackling critical scientific challenges [4]. Notably,large language models (LLMs) [5] have demonstrated that model performance improves substantially with larger datasets and model size, following empirical scaling laws [6]. Models like GPT-4 derive much of their effectiveness from access to massive public datasets. However, high-quality private data, such as medical and user behavior data [7,8], are often isolated among clients, making centralized training infeasible.

Federated learning (FL) addresses this challenge by enabling decentralized training over private data, allowing clients to improve a shared model without centralizing sensitive information [9]. FL has been successfully applied in privacy-sensitive domains, including healthcare [10], the Internet of Things [11], and personalized recommendations [12]. However, although FL avoids direct data sharing, model updates themselves can still leak sensitive information. Recent studies have shown that shared gradients or model updates may expose private data through gradient inversion and related attacks [13]. This limitation motivates the need for secure-aggregation mechanisms that protect local updates while still allowing effective collaborative training.

Meanwhile, quantum machine learning has emerged as an active research direction that combines quantum theory and machine learning and introduces new learning models and paradigms for quantum information-processing platforms [14–16]. Building on this direction, quantum federated learning (QFL) [17] extends FL to settings where clients may access quantum resources or use quantum and hybrid quantum-classical models during local training. In this sense, QFL naturally connects the decentralized-learning framework of FL with the emerging learning paradigms of quantum machine learning.

However, QFL does not by itself resolve the privacy risks associated with model-update sharing. Recent protocols [18–22] have been proposed to improve communication efficiency in QFL, using quantum machine-learning models like quantum neural networks (QNNs) [23], which are promising for the noisy intermediate-scale quantum era. While communication efficiency is critical, ensuring data privacy remains the primary concern in QFL. Recent efforts have attempted to mitigate these risks through sophisticated quantum algorithms [24–29] or by incorporating differential privacy techniques [30]. However, many of these approaches require extensive quantum resources beyond current capabilities or do not provide information-theoretic privacy guarantees for model-update aggregation. Consequently, there remains a strong need for a practical QFL protocol that offers quantum-level security for privacy-preserving aggregation in the coming era of large-scale quantum computing.

To address this challenge, a natural direction is to consider security primitives provided by quantum networks. Quantum key distribution (QKD), as a foundational primitive of the quantum internet [31], enables confidential key generation with information-theoretic security. With major experimental progress in recent years [32–39], QKD provides a realistic basis for secure aggregation in QFL settings, where both privacy and scalability are important [40]. More broadly, the rapid development of quantum networks [31,41–44] and recent demonstrations of quantum-network capabilities [45–51] further motivate the study of practical privacy-preserving learning protocols built on quantum communication infrastructure.

**Fig. 1.**
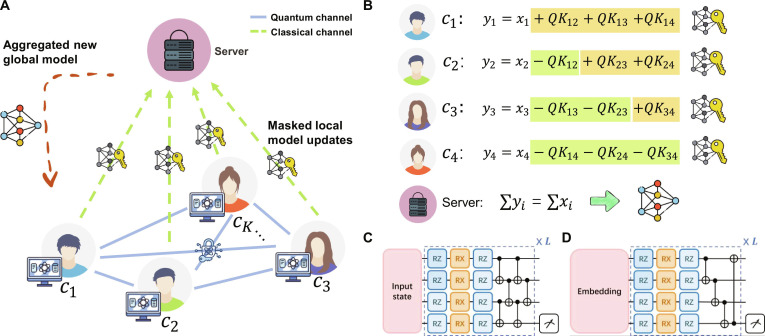
Schematic of the QuNetQFL. (A) The QuNetQFL employs a fully connected quantum network among K clients, where each client uses quantum key distribution (QKD) to securely exchange quantum secure keys with others, enabling pairwise masking of local model updates. Only these masked updates are sent to the server through classical channels, preserving client privacy. The server aggregates the updates to reconstruct the global model parameter, which is then redistributed to all clients. The minimal quantum resource requirement for all clients is access to a fully connected QKD network. (B) Masked secure aggregation in a 4-client setup. Here, $x_{i}$ represents the raw local updates and $y_{i}$ denotes the masked updates. The server sums the masked updates to obtain the decrypted global model update. (C and D) Two types of quantum neural network (QNN) representations with hardware-efficient ansatzes used in the study. RX, rotation gate around the *x*-axis; RZ, rotation gate around the *z*-axis.

In this work, we propose QuNetQFL, a practical QFL protocol natively implemented on quantum networks, ensuring secure aggregation of model updates with information-theoretic security. Unlike QFL schemes that rely on costly homomorphic encryption [29,52,53] or complex encrypted multi-qubit states preparation and transmission [24–27], QuNetQFL utilizes secure keys generated by QKD to create pairwise masks for client updates (see Fig. 1 for an illustration). Compared to classical secure-aggregation schemes [54], our approach realizes information-theoretic one-time-pad masking using QKD-derived keys and, to the best of our knowledge, provides the first experimentally validated quantum-secure FL protocol deployed on a multi-user quantum network. Specifically, we validate our protocol by employing 4-phase measurement-device-independent (MDI) QKD [36] in a 4-client quantum network. Using a Sagnac interferometer for phase stability, we present a proof-of-principle demonstration of a multi-user quantum network over 6 km of optical fiber, achieving a secret-key rate exceeding 32.8 kbps. This experimental demonstration confirms that near-term quantum networks can already support practical, scalable distributed learning tasks.

Leveraging experimentally generated quantum keys, we comprehensively benchmark QuNetQFL across a range of scenarios, each utilizing different quantum resources, from quantum, hybrid quantum-classical, to classical models, and quantum to classical datasets. These benchmarks include (a) classification of 2 quantum datasets using QNNs, (b) sentiment analysis using a hybrid classical-quantum LLM (BERT–QNN), and (c) handwritten-digit recognition using QNNs. In all cases, QuNetQFL demonstrates remarkable performance. We show that adding a quantum client substantially enhances the global model’s ability to classify multipartite entangled and quantum magic datasets, resulting in at least a 2% improvement in test accuracy and a reduction in loss, which highlights the protocol’s scalability and robustness. In sentiment analysis, our scheme supports collaborative fine-tuning of BERT–QNN across multiple clients, using widely recognized IMDb, Yelp, and Amazon review datasets [55]. This is validated on both simulated and real quantum hardware, demonstrating the effectiveness of QuNetQFL in enhancing LLM training. For handwritten-digit recognition on the Modified National Institute of Standards and Technology (MNIST) dataset, we achieve comparable accuracy (within 1%) under both nonindependent and identically distributed (non-IID) and IID data distributions. Further simulations highlight QuNetQFL’s scalability to 200 clients, using classical learning models, LeNet-5, with rapid convergence and an at most 75% reduction in communication cost facilitated by advanced model compression techniques. Overall, our work addresses the critical need for a practical QFL framework that efficiently operates within quantum hardware limitations, ensuring quantum security from the present day through to the era of large-scale quantum computing.

## Results

### Framework of QuNetQFL

We consider a fundamental QFL setting with one central server and K quantum-enabled clients. Client k holds a local dataset $D_{k}={\left\{\left(\rho_{k}^{i},y_{k}^{i}\right)\right\}}_{i=1}^{n_{k}}$, where $\rho_{k}^{i}$ is a quantum state and $y_{k}^{i}$ is the corresponding label. Datasets can be native quantum data or classical data encoded into quantum states $\rho \left(x_{k}^{i}\right)$ [56]. In communication round t, the server broadcasts global QNN parameters $\theta^{t-1}\in {\mathbb{R}}^{M}$. Each client then trains a local model $\theta_{k}^{t}$ by minimizing a local loss $L\left(\theta_{k}^{t}\right)$ and returns the update $\Delta \theta_{k}^{t}=\theta_{k}^{t}-\theta^{t-1}$. The server aggregates updates via a weighted sum

$$\Delta \theta^{t}=\sum_{k=1}^{K}\frac{n_{k}}{N}\;\Delta \theta_{k}^{t},\;N=\sum_{k=1}^{K}n_{k},$$ (1)

and updates the global model $\theta^{t}=\theta^{t-1}+\Delta \theta^{t}$. While no raw data leave clients, sharing gradients or updates is known to be vulnerable to inversion attacks. Ensuring privacy therefore requires encryption methods that remain secure even in the presence of quantum computers. We thus adopt a quantum-enhanced secure-aggregation protocol utilizing QKD-based masking, namely, QuNetQFL, to achieve information-theoretic security.

### Quantum-secure aggregation via QKD masking

QuNetQFL uses pairwise quantum secret keys distributed over a fully connected quantum network to one-time-pad mask local updates (Fig. 1B). In round t, the server selects clients $S^{t}\subseteq \left\{1,\ldots ,\;K\right\}$ randomly. Each selected pair $\left(i,j\right)$ shares a q-bit key vector ${\mathrm{QK}}_{i,j}^{t}\in {\mathbb{Z}}^{M}$ (symmetric: ${\mathrm{QK}}_{i,j}^{t}={\mathrm{QK}}_{j,i}^{t}$). To make efficient use of these keys, model updates are quantized to q bits before masking (quantizer and de-quantizer are defined in Methods). By choosing q appropriately, this quantization not only enables effective masking but also reduces both key consumption and communication overhead.

Each client uploads a masked, quantized update

$$\Delta {\tilde{\theta }}_{i}^{t}=\left[\;Q^{q}\;\left(p_{i}^{t}\cdot \Delta \theta_{i}^{t}\right)+m_{i}^{t}\;\right]\text{mod}\;2^{q},$$ (2)

where $Q^{q}\left(\cdot \right)$ denotes q-bit quantization, $p_{i}^{t}$ is the weight proportional to the client’s data size, and $m_{i}^{t}$ is the masking term constructed as a signed sum of pairwise QKD keys shared between client i and the other participants. When all updates are summed, the masks cancel out (see Methods for details), leaving only the aggregated, quantized update.

$$\Delta \theta^{t}=\left[\sum_{i\in S^{t}}Q^{q}\;\left(p_{i}^{t}\cdot \Delta \theta_{i}^{t}\right)\right]\text{mod}\;2^{q}.$$ (3)

The server then reconstructs the global model by de-quantization,

$$\theta^{t}=\theta^{t-1}+D^{q}\;\left(\Delta \theta^{t}\right),$$ (4)

where $D^{q}\left(\cdot \right)$ reverses quantization. This protocol ensures that only the aggregated update is revealed, while individual contributions remain hidden. Key cost grows as $O\left(K^{2}M\right)$ with client number K and model size M, but is substantially reduced by q-bit quantization and, at larger scale, by model compression. Full protocol details and pseudocode are provided in Methods (Algorithm 1).

This quantum-secured aggregation approach provides perfect privacy against gradient attacks, supporting secure and scalable QFL across distributed networks. The theoretical convergence analysis for QuNetQFL is provided in the Supplementary Materials, offering convergence guarantees and practical guidance for parameter selection and training.

### Experimental quantum secret-key generation

To ensure an adequate supply of quantum secret keys, we implemented the 4-phase MDI-QKD [36] across 3 and 4 clients within a quantum network, with each client pair generating keys over a 200-s duration. These quantum keys were then used to secure several collaborative learning tasks demonstrated in the following section, with local model updates encrypted before aggregation. A Sagnac loop was employed to stabilize the phase, and a 6-km optical fiber was inserted into the loop.

In the 3-client scenario, the accumulated keys were sufficient to support training a model with up to 1,434 parameters, represented as 32-bit floating-point values, over 200 communication rounds. In the 4-client case, the number of supported parameters decreased slightly to 1,393 due to the additional pairwise connections increasing the demand on the key pool. The secure key rates achieved for different client pairs are summarized in Table 1. Further details of the MDI-QKD implementation, including experimental setup and simulation, are provided in Methods.

**Table 1.** Summary of key rates (bits per second, bps). A, B, C, and D represent Alice, Bob, Charlie, and David, respectively.

3-client		4-client			
Client pair	Key rate (kbps)	Client pair	Key rate (kbps)	Client pair	Key rate (kbps)
AB	230	AB	240	BC	44.6
AC	37.6	AC	35.6	BD	36.6
AD	45.9	AD	43	CD	32.8

Although we employ MDI-QKD here, the protocol can be substituted by other QKD schemes depending on practical requirements, serving as a proof-of-principle implementation of QFL. Since secret keys can be pre-generated and stored, the time required for key generation is not a limiting factor in performance. Consequently, our framework is highly flexible and exhibits strong compatibility with emerging quantum communication infrastructures, including satellite-based QKD links, trusted-node architectures, and entanglement-based networks, paving the way toward scalable and secure distributed learning in the quantum era.

### Classification of quantum datasets using QNNs

Detecting and certifying quantum resources, such as entanglement and quantum magic (nonstabilizerness), are essential for advancing practical quantum technologies [57–60]. To tackle this, we employed QuNetQFL to classify 2 quantum datasets: one with multipartite entangled states (high vs. low entanglement) and the other with quantum magic states (nonstabilizer vs. stabilizer). We performed these classifications under an IID data distribution setting, utilizing QNNs based on a hardware-efficient ansatz (HEA) [61]. Comprehensive details of the datasets and simulations are provided in the Methods.

**Fig. 2.**
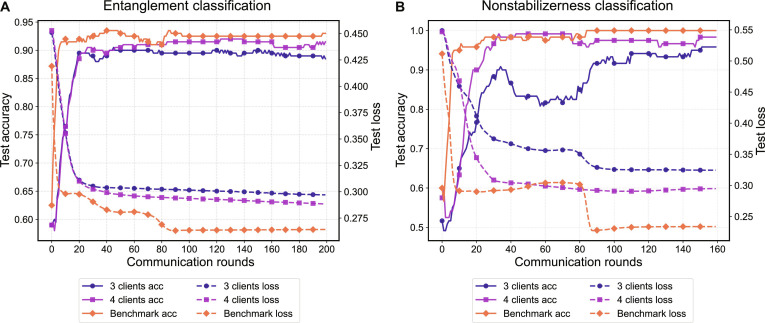
Performance of QuNetQFL on quantum state classification using QNN. (A) Entanglement classification and (B) nonstabilizerness classification results across different client setups. Adding a single client notably brings the model accuracy and training convergence closer to the benchmark.

By utilizing experimentally generated secret keys for quantum-secure aggregation, we demonstrated secure collaborative learning capabilities across 3-client and 4-client scenarios. Figure 2 compares the performance of these scenarios, using 16-bit quantization (q=16), against an ideal benchmark where all client data are centrally processed. Results show that the final test accuracies for classifying high versus low entangled states are 88.5% with 3 clients, 91.5% with 4 clients, and 93% for the benchmark. For nonstabilizer versus stabilizer states classification, the final test accuracies are 95.8% with 3 clients, 98.3% with 4 clients, and 100% for the benchmark. In both datasets, the results show a substantial improvement in the global model’s performance, with at least a 2% improvement in test accuracy of the 4-client setting over the 3-client setting, together with a reduction in loss. The addition of a single client in both classification tasks brings the model closer to the benchmark, emphasizing the protocol’s scalability and potential for secure, collaborative learning across multiple quantum clients, each operating its own QNN and serving as an individual quantum data center [62].

### Sentiment analysis using hybrid BERT–QNN

**Fig. 3.**
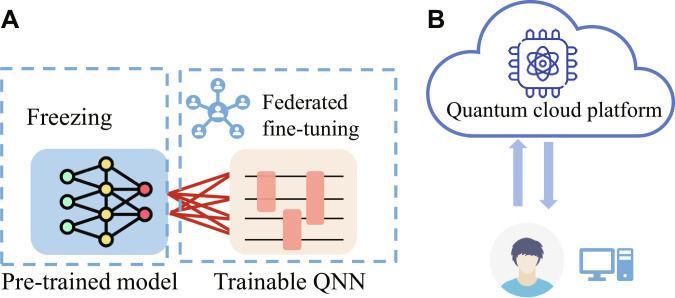
Schematic of federated fine-tuning the hybrid BERT–QNN model. (A) Multiple clients collaboratively fine-tune a hybrid BERT–QNN model on specific datasets, such as sentiment analysis tasks. The pre-trained BERT model is frozen, and only the parameters of the QNN (orange) and the connecting linear layer (red) are efficiently fine-tuned through the QuNetQFL protocol. (B) The trained hybrid model is evaluated on a superconducting quantum computing cloud platform, with performance metrics derived from real quantum hardware evaluations.

To further evaluate the scalability and hardware relevance of QuNetQFL beyond simulations, we apply our protocol to a series of 2-class language-classification tasks, meaning classification (MC) and relative pronoun (RP), 2 simple synthetic datasets introduced from Ref. [63], and 3 sentiment analysis benchmarks from real world (Yelp, IMDb, and Amazon). These tasks are tackled by federated fine-tuning the quantum subcircuit of a hybrid BERT–QNN model (see Fig. 3 for an illustration). In this simulation, 4 clients participate, each receiving an equal share of the training examples in an IID data partition. The quantum subcircuit consists of a 4-qubit 3-layer QNN with a HEA. Further simulation details are provided in the Methods and Supplementary Materials.

**Fig. 4.**
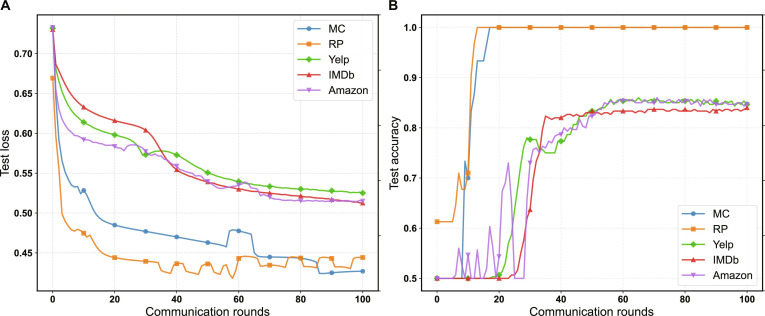
Sentiment analysis through hybrid BERT–QNN. (A) Training loss curves over 100 epochs for 5 language-classification tasks on meaning classification (MC), relative pronoun (RP), Yelp, IMDb, and Amazon, using a 4-qubit, 3-layer hardware-efficient ansatz QNN. All models are fine-tuned via QuNetQFL with secure aggregation and quantized updates. The loss rapidly decreases within the first 50 rounds. (B) Corresponding test accuracy curves showing fast convergence. RP and MC converges near-perfect accuracy by 20 rounds, while the sentiment tasks stabilize above 84% accuracy.

Figure 4 shows the simulation results of test accuracy and loss curves for these 5 language-classification tasks. These results underscore QuNetQFL’s adaptability to hybrid classical-quantum architectures. To further validate end-to-end operation on real quantum hardware, we transpile the trained quantum circuits for these tasks into the device’s native gate set and run them on the BAQIS Quafu quantum computing cloud (156-qubit superconducting quantum chip with a median 1Q error of 7.6 × 10^−4^ and a median 2Q error of 1.8 × 10^−2^), averaging from 4,000 shots per circuit evaluation. Table 2 compares the final test accuracy between numerical simulations and real quantum hardware. For synthetic datasets MC and RP, both simulated and quantum hardware results achieved 100% accuracy. The quantum hardware slightly outperformed the simulated result on the Yelp dataset, while an accuracy degradation of 0.7% and 3.7% was observed on IMDb and Amazon, respectively. These results demonstrate the robustness of the trained model to noise on real quantum devices using our method.

**Table 2.** Final test accuracy of BERT–QNN and the dimension-matched classical baseline

Task	BERT–QNN sim.	BERT–QNN hardware	Classical baseline
MC	1.000	1.000	1.000
RP	1.000	1.000	1.000
Yelp	0.847	0.850	0.840
IMDb	0.840	0.833	0.820
Amazon	0.847	0.810	0.817

To provide a controlled classical reference, we further evaluated a dimension-matched classical baseline under the same frozen BERT encoder, 4-client IID partition, 100-round QuNetQFL protocol, 16-bit quantization, and QKD-key-based secure aggregation. In this baseline, the 768-dimensional BERT representation is reduced to a 4-dimensional feature vector and then processed by a lightweight classical nonlinear classification head. As shown in Table 2, the BERT–QNN model achieves comparable final-round performance to this classical baseline, with identical accuracy on MC and RP and slightly higher simulation accuracy on Yelp, IMDb, and Amazon. This comparison is intended as a controlled classical reference rather than evidence of quantum advantage.

### Handwriting recognition by QNNs

**Fig. 5.**
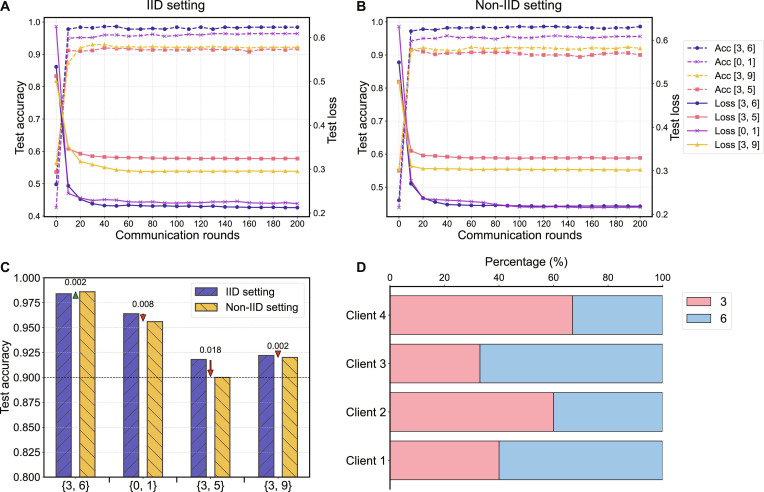
Evaluation of QuNetQFL on the MNIST classification task using QNN. (A) Independent and identically distributed (IID) setting showing test accuracy and loss across 200 communication rounds for client combinations {3, 6}, {0, 1}, {3, 5}, and {3, 9} with 16-bit quantization. (B) Non-IID setting with identical client configurations as (A) for comparative analysis. (C) Final test accuracy comparison between IID and non-IID settings, with annotated accuracy differences illustrating QuNetQFL’s robustness across data distributions. (D) Non-IID client data distribution proportions for classes {3, 6} (same for other cases), highlighting dataset heterogeneity.

To demonstrate the robustness and adaptability of QuNetQFL in real-world, heterogeneous data environments, we evaluated its performance across both IID and non-IID settings for 4 clients using QNNs on the classical MNIST dataset. We created multiple 2-class subsets from MNIST, specifically {3, 6}, {0, 1}, {3, 9}, and {3, 5}, where, for instance, {3, 9} represents images of digits “3” and “9”. Each client was allocated 500 training samples from the MNIST training set, while the server test set comprised 500 samples from the MNIST test set. In the IID setting, each client’ data contained an equal number of samples from each category. In contrast, the non-IID setting introduced imbalanced distributions to better reflect real-world scenarios where data vary across devices, presenting greater challenges for FL effectiveness. The data splits among the 4 clients for 2 classes were configured as (200, 300), (300, 200), (167, 333), and (333, 167), as shown in Fig. 5D. To accommodate quantum resource constraints, we resized the original 28 × 28 images to 4 × 4 and encoded each into 4-qubit states via amplitude embedding. The classification task utilized a 4-qubit QNN with a 3-layer HEA, as shown in Fig. 1D. Results in Fig. 5 show rapid convergence of the test loss within the first 40 communication rounds for both IID and non-IID settings, as depicted in Fig. 5A and B. A comparison of final test accuracies across both data distributions, as shown in Fig. 5C, reveals close performance levels (within 1% test accuracy) with a close communication time (≈40 rounds), highlighting the strong adaptability of our QFL protocol across varying data-distribution conditions. Details of the hyperparameter settings are provided in the Supplementary Materials.

### Simulation of the large-scale implementation of QuNetQFL

The QuNetQFL protocol is flexible, supporting both quantum and classical machine-learning models. If quantum computational resources are unavailable on either the clients or server, classical models can be used, ensuring scalability while benefiting from quantum-secure communication at scale, particularly in quantum metropolitan area networks.

To evaluate the scalability of QuNetQFL, we implemented it using the classical LeNet5 model (61,706 parameters) and the MNIST dataset in an IID setting. The training set was equally divided among 200 clients, each receiving 300 instances randomly. In each aggregation round, the server randomly selected 5% of the clients. The total number of rounds was set to 200, and clients performed local training with 5 local epochs, a batch size of 32, and the Adam optimizer at a learning rate of 0.01. Due to limitations in our ability to implement such scale quantum networks, we used pseudorandom numbers as masks instead of true quantum secret keys in this experiment to illustrate the impact of quantization on reducing quantum secret key costs in QuNetQFL.

Table 3 presents results for q=8,16,32-bit quantization and plaintext aggregation (benchmark), showing the quantum secret key cost (related to model update size) and corresponding test accuracy. The results show that 8-bit quantization reduces the quantum key cost by 4×, and 16-bit quantization achieves a 2× reduction compared to 32-bit quantization in each communication round. Despite 200 clients, convergence occurred within 20 to 40 rounds, with consistent performance across quantization levels and benchmarks, leading to substantial communication savings, lower key costs, and reduced time in real-world collaborative learning. This evaluation demonstrates the role of quantization techniques employed in the QuNetQFL for balancing model performance and communication efficiency for scalable quantum-enhanced FL.

**Table 3.** Cost of quantum secret keys and test accuracy for 200 clients under different quantization levels

	Cost (MB)	Accuracy
32-Bit quantization	10.593	0.9798
16-Bit quantization	5.296	0.9738
8-Bit quantization	2.648	0.9704
Benchmark	–	0.9860

–, not applicable.

## Discussion

In this work, we introduce QuNetQFL, a QFL protocol leveraging quantum networks to achieve information-theoretic security during model updates. By employing QKD, we ensure encrypted communication among clients, where local model updates are protected by quantum secret keys and masked via one-time pads. The protocol also incorporates efficient quantization techniques and is adaptable to other compression methods, such as low-rank tensor compression, weight pruning, and knowledge distillation, to further reduce both communication and quantum key costs.

We experimentally validated QuNetQFL on a 5-node quantum network, achieving quantum-secret-key generation with key rates exceeding 30 kbps in both 3-client and 4-client scenarios. Notably, protocols such as twin-field QKD, based on the Sagnac loop used in our experimental scheme, can reach distances of up to 200 km [64], suggesting the potential for large-scale QFL applications in metropolitan quantum networks. Our protocol demonstrates that near-term quantum networks can already support realistic FL tasks, offering a platform for scaling distributed learning systems in quantum networks.

Through both simulations and hardware-based evaluations, we demonstrate QuNetQFL’s broad applicability across diverse tasks, including quantum resource classification, sentiment analysis, and handwritten-digit recognition. These tasks were executed using quantum, classical-quantum hybrid, and classical learning models, each corresponding to different levels of quantum computing requirements. Notably, we fine-tuned a hybrid classical-quantum LLM within our protocol and evaluated it on real quantum hardware for various language-classification tasks. The results showed robust performance, comparable to those obtained in simulation, underscoring the practical viability and scalability of QuNetQFL. This validation extends beyond simulation-based studies, confirming that the protocol can effectively operate in real-world scenarios, even within the context of LLMs.

While this work does not demonstrate a quantum advantage in computational or communication complexity, it provides a practical solution for quantum-secure FL. Approaches such as differential privacy degrade model accuracy by introducing noise [30], while schemes based on homomorphic encryption or post-quantum cryptography with blockchain [55,65] offer computational security, but rely on unproven hardness assumptions and impose substantial computational and communication overheads. Other protocols offering unconditional security, like entanglement-based secure aggregation [24,27], require quantum resources beyond current capabilities, and gradient-hiding methods [25] suffer from circuit complexity and accuracy loss. In contrast, QuNetQFL achieves information-theoretic security through QKD-based masking, requiring only pairwise secret key—resources already available in current quantum networks, making it a practical and near-term route to secure and scalable QFL. We note that the secure-aggregation workflow of QuNetQFL is modular with respect to the key-establishment mechanism. In principle, classical or post-quantum key-establishment methods could also be used to generate the pairwise keys required for cancelling-mask construction, uploading masked local updates, and server-side aggregate recovery. However, such substitutions would change the security properties: QKD-derived keys enable information-theoretic security, whereas classical and post-quantum alternatives provide computational security based on the assumed hardness of the underlying cryptographic primitives.

Additionally, blind quantum computing presents a promising approach to quantum privacy computing, which can seamlessly integrate with our protocol. Blind quantum computation enables clients with minimal quantum resources to delegate tasks to an untrusted server while keeping the input, algorithm, and output confidential [30,66]. However, existing schemes do not inherently protect the FL aggregation phase, where model updates can be vulnerable to gradient-leakage attacks. While differential privacy is used in prior works [30] to protect this phase, it incurs a trade-off in model utility. Our QuNetQFL protocol can replace differential privacy in these schemes, offering information-theoretic security without compromising model accuracy. A detailed algorithm and discussion are provided in the Supplementary Materials. This enhancement positions QuNetQFL to integrate with experimental advancements in multi-user blind quantum computing [66], further advancing the practicality of scalable QFL.

Future work will focus on reducing communication complexity by integrating these advanced quantum algorithms, balancing efficiency with practical implementation. Additionally, there is an emerging trend to combine QKD and post-quantum cryptography to harness the strengths of both, enhancing the security and efficiency of quantum communication systems [67,68]. We believe this approach holds great potential for building privacy-preserving distributed learning systems in the future. Although QuNetQFL was primarily evaluated on variational quantum circuit models for the noisy intermediate-scale quantum era, it is also adaptable to large-scale quantum machine-learning models [69], making it relevant for future fault-tolerant quantum computing. We anticipate that this work will stimulate the development of more practical and scalable QFL schemes, further advancing quantum technologies in distributed learning systems.

## Materials and Methods

The detailed protocol for QuNetQFL is provided in Algorithm 1. Importantly, QuNetQFL is designed to be flexible, allowing clients to choose their local training algorithms based on resource constraints and computational requirements. Clients can employ either gradient-based methods or alternative non-gradient-based methods to update local models, particularly when obtaining the gradients of the QNN circuit is expensive.


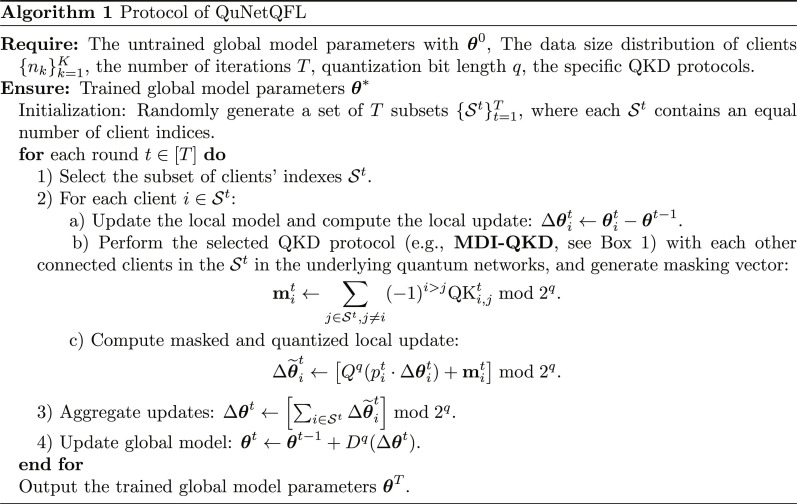

Box 1. MDI-QKD Protocol subroutine

 **for** Each client $\left(i,\;j\right)$ pairs in $S^{t}\times S^{t}$ do

1) Randomly choose basis (X or Y) and prepare corresponding weak coherent states ${⊗}_{k=1}^{n}|e^{{\mathrm{i\varphi}}_{k}}\sqrt{\mu }\rangle$, where μ is the pulse intensity.
2) Send the states to an untrusted node, Eve, for measurement.
3) Decide whether to flip the bit according to the measurement outcome.
4) Estimate bit error rate by clients’ announcements.
5) Perform postprocessing steps (error correction and privacy amplification) to generate enough final secure keys.

 **end for**

**Note:** Secret keys used in QuNetQFL can be generated during training or pre-generated to the overall process time. MDI-QKD reduces the minimal quantum resources required for client participation, and QuNetQFL is flexible to emerging QKD techniques.

### QKD-based masking for secure aggregation

In this subsection, we outline the technical details of secure aggregation using QKD-based masking. In each round, client $i\in S^{t}$ constructs a mask

$$m_{i}^{t}=\sum_{j\in S^{t},j\neq i}{\left(-1\right)}^{i>j}\;{\mathrm{QK}}_{i,j}^{t}\;\text{mod}\;2^{q},$$ (5)

where ${\mathrm{QK}}_{i,j}^{t}$ is the pairwise quantum key shared by clients i and j. The alternating signs ensure that masks cancel exactly when summed over all participating clients. Client i then uploads the masked, quantized update

$$\Delta {\tilde{\theta }}_{i}^{t}=\left[\;Q^{q}\left(p_{i}^{t}\cdot \Delta \theta_{i}^{t}\right)+m_{i}^{t}\;\right]\text{mod}\;2^{q},\;p_{i}^{t}=\frac{n_{i}}{\sum_{\ell \in S^{t}}n_{\ell }},$$ (6)

where $Q^{q}\left(\cdot \right)$ is the q-bit quantization function and $p_{i}^{t}$ is the client weight. Here, each ${\mathrm{QK}}_{i,j}^{t}$ is represented as an M-entry vector of q-bit words (equivalently, an $\left(M\times q\right)$-bit string), with M the model size. Each entry is used as two’s-complement signed integer representation in $\left[-\left(2^{q-1}-1\right),\;2^{q-1}-1\right]$, and all masking and aggregation are carried out as additions in ${\mathbb{Z}}_{2^{q}}$, rather than the bitwise exclusive OR (XOR) commonly used with QKD keys in quantum cryptography, to match the q-bit quantized updates.

The server aggregates masked updates by

$$\Delta \theta^{t}=\left[\sum_{i\in S^{t}}\Delta {\tilde{\theta }}_{i}^{t}\right]\text{mod}\;2^{q}$$ (7)

$$=\left[\sum_{i\in S^{t}}Q^{q}\left(p_{i}^{t}\cdot \Delta \theta_{i}^{t}\right)\right]\text{mod}\;2^{q},$$ (8)

where the cancellation property

$$\left[\sum_{i\in S^{t}}m_{i}^{t}\right]\text{mod}\;2^{q}=0$$ (9)

ensures that individual masks cancel out and the quantized updates are successfully decrypted.

### Quantization technique

To efficiently utilize the secret keys generated in quantum networks and further reduce the communication overhead, we employ a quantization technique in QuNetQFL, originally developed for homomorphic encryption [53] and later adapted for secure aggregation [70]. This method quantizes a scalar s∈ℝ within $\left[-\beta ,\;\beta \right]$ into a q-bit sign integer in the range $\left[-\left(2^{q-1}-1\right),\;2^{q-1}-1\right]$ using the q-bit quantizer $Q^{q}\left(s\right)$

$$Q^{q}\left(s\right)=\text{sgn}\left(s\right)\cdot \text{Round}\left(\mathrm{abs}\left(s\right)\cdot \left(2^{q-1}-1\right)/\beta \right),$$ (10)

where $\text{sgn}\left(\cdot \right)$ is the sign function, $\mathrm{abs}\left(\cdot \right)$ denotes the absolute value, and $\text{Round}\left(\cdot \right)$ maps the input to the nearest integer. The corresponding de-quantized process for a quantized value v is given by

$$D^{q}\left(v\right)=\text{sgn}\left(v\right)\cdot \left(\mathrm{abs}\left(v\right)\cdot \beta /\left(2^{q-1}-1\right)\right).$$ (11)

Quantization is performed on the client side for local model updates, while de-quantization is executed on the cloud server for the aggregated global model. Model parameters are clipped to the range $\left[-\beta ,\beta \right]$ to ensure quantization accuracy. Note that to handle signs during de-quantization, the server employs the following adjustment: If $v>2^{q-1}-1$, it is updated as $v-2^{q}$; otherwise, v remains unchanged.

In practice, β is selected according to the dynamic range of the quantity being quantized. For global QNN parameters, which correspond to periodic circuit rotation angles naturally bounded to $\left[-\pi ,\;\pi \right]$ by the periodicity of quantum gates, we set β=π. For local model update differences, β must additionally prevent integer overflow during modular aggregation in ${\mathbb{Z}}_{2^{q}}$ before de-quantization since the server reconstructs the aggregated quantized update from $N_{c}$ participating clients rather than individual updates. We therefore conservatively set $\beta =N_{c}\beta_{0}$, where $N_{c}$ is the number of selected clients and $\beta_{0}$ bounds the typical per-client update magnitude. In our experiments, $\beta_{0}=1$ for the QNN tasks and $\beta_{0}=0.1$ for the BERT–QNN fine-tuning tasks, reflecting the smaller update magnitudes in the latter setting. These values were empirically verified in the reported training runs to avoid excessive clipping while maintaining stable training accuracy.

### Security analysis

(a) Threat model. In our framework, we adopt the standard *honest-but-curious* setting in FL, where clients and the server follow the protocol but may attempt to infer others’ data from messages. We exclude the extreme (and impractical) case where the server colludes with all but one client during a secure sum [54]. This *honest-but-curious* assumption also excludes attacks from malicious clients during quantum key exchanges, such as denial-of-service attacks. Using a star-topology QKD network for key distribution could mitigate such risks and move beyond the *honest-but-curious* assumption, representing a valuable direction for future work. (b) Security guarantee. QKD enables the distribution of unconditional secure keys between 2 distant parties. By using pairwise QKD keys as one-time pads in the q-bit quantized domain, the uploaded vector $\Delta {\tilde{\theta }}_{i}^{t}$ becomes information-theoretically indistinguishable from random to any party lacking the corresponding keys. The server learns only the aggregate (after mask cancellation), never an individual client’s update. Our guarantees protect client data against other clients, the server, and external adversaries. However, protecting the global model is not the goal of this work.

### Secret-key distribution in experimental quantum network

**Fig. 6.**
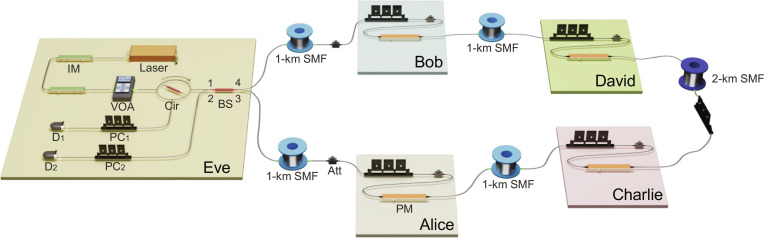
Experimental setup of the quantum network. At Eve’s site, a continuous-wave laser source is employed with 2 intensity modulators (IMs) and a variable optical attenuator (VOA) to produce weak coherent pulses. Four clients (Alice, Bob, Charlie, and David) are interconnected in the Sagnac loop. When any pair of clients need to establish secret keys, Eve injects the pulses into the loop via a circulator (Cir) and a 50:50 beam splitter (BS). Upon reaching the designated clients, phase modulators (PMs) are employed to add phase to the pulses. After modulation, the 2 pulse trains interfere at Eve’s BS, where interference results are detected by 2 superconducting nanowire single-photon detectors (D_1_ and D_2_). This structure includes 1-km single-mode fibers (SMFs) between Alice and Eve, Bob and Eve, Alice and Charlie, and Bob and David, while a 2-km SMF connects Charlie and David. Attenuators (Att) are placed between Alice and Eve, and between Bob and Eve, to adjust channel losses. Polarization controllers (PCs) are used to align polarization in the loop.

To experimentally generate quantum secure keys for demonstrating QuNetQFL with 3 or 4 clients, we established a quantum network with 5 participants. This includes 4 clients, Alice, Bob, Charlie, and David, who potentially require quantum secret keys for collaborative training, and one party, the untrusted Eve, as shown in Fig. 6. In this setup, Eve is responsible for measuring interference results and sending unmodulated coherent pulses as the coherent state source. This can be understood as the “quantum channel” in Fig. 1A at an abstract level. Through this quantum network, each client pair can securely share quantum secret keys.

Here, we take the key generation process of MDI-QKD between Alice and Bob as an example, which applies similarly to other client pairs. The system frequency is 100 MHz. The relevant protocol description can be found in Methods. In Eve’s site, to prepare weak coherent pulses with an extinction ratio exceeding 30 dB, 2 intensity modulators are utilized to chop the continuous light from a narrow-linewidth continuous-wave laser source (NKT Photonics E15 erbium fiber laser). Eve then sends the pulses into the loop after passing through a circulator and a 50:50 beam splitter (BS). In the loop, 2 clients modulate the pulse train with phase modulators. Note that users only modulate one pulse train. Specifically, Alice only modulates the clockwise pulses and Bob only modulates the counterclockwise pulses. Then, the 2 pulses interfere at Eve’s BS, and Eve records corresponding detection events by D_1_ and D_2_. The detection efficiencies of D_1_ and D_2_ are 60.3% and 64.5%, respectively, with dark count rates of approximately 18 and 28 Hz. The time-window length is set to 1.8 ns. A time window is a fixed-duration interval within each pulse period, during which detection events are considered valid, while detection events outside this window are discarded. For Alice and Bob, we adjusted the attenuation values by attenuators between the users to make the total channel loss 6 dB, while for other client pairs, the total channel loss is set to 12 dB. The attenuation values are adjusted to ensure the channels are symmetric.

Our network design offers enhanced scalability and cost efficiency for QKD. In this setup, a Sagnac loop is employed to stabilize the phases among different users. The laser source and single-photon detectors are positioned at the party, Eve, outside the loop. Regardless of the number of clients, only one Eve is required for detection, thereby reducing the demand for measurement devices when performing MDI-QKD. Clients in this structure are only required to perform phase modulation on weak coherent states, eliminating the need for complex quantum state preparation and measurement. This design also allows for easy adjustment of the number of users. Adding or removing a user does not require any modifications to the detection terminal. For users already in the network, when they need to begin the key generation process, they only need to increase or decrease the fiber length to avoid collisions of pulses in both clockwise and counterclockwise directions.

The probability of choosing the X basis is 90% during implementation, and the period length of random number is 10,000. Error rates varied across different user pairs, prompting us to use a genetic algorithm to optimize the key rate for each pair under these differing error conditions. This optimization led to variations in pulse intensity across pairs.

### Protocol of quantum network and its simulation details

We employed a 4-phase MDI-QKD protocol [36] between each pair of participants to generate quantum secret keys. To illustrate this process, we use the example of Alice and Bob with an untrusted third party, Eve. All other user pairs in the network follow this same protocol to obtain their quantum secret keys.

1. Alice and Bob choose the X basis with the probability $p_{x}$ and the Y basis with the probability $p_{y}$ ($p_{y}=1-p_{x}$). For the X basis, they prepare $|e^{ik_{x}\pi }\sqrt{\mu }\rangle$ where $k_{x}$ is the logic bit value ($k_{x}\in \left\{0,1\right\}$) and μ is the pulse intensity. For the Y basis, they prepare $\left|e^{i\left(k_{y}+1/2\right)\pi }\right.\sqrt{\mu }\rangle$ where $k_{y}$ is the logic bit value ($k_{y}\in \left\{0,\;1\right\}$).
2. Eve performs measurements on the pulses with a 50:50 BS and 2 single-photon detectors and records the detection results. One and only one detector clicks is defined as an effective event.
3. The above steps are repeated many times to accumulate sufficient data. Eve announces all effective events and the corresponding detector that clicks. For each effective event announced by Eve, if D_2_ clicks, Bob flips his corresponding logic bit. Alice and Charlie retain only the logical bits from effective measurements, discarding others. Then they disclose their basis choices for effective events through authenticated classical channels.
4. Alice and Bob announce all their bit values in the Y basis to calculate the quantum bit error rate $E_{b}^{y}$ in the Y basis to estimate the phase error rate under the X basis $E_{p}$, and the number of counts $n_{x}$ and $n_{y}$ can also be obtained under X and Y bases, respectively.
5. Alice and Bob perform error correction on the remaining keys under the X basis and privacy amplification to obtain the final secret keys.

The final key rate of MDI-QKD [36] can be given by

$$l=\;n_{x}\left[1-H\left({\bar{E}}_{p}\right)\right]-\lambda_{EC}-{\text{log}}_{2}\frac{2}{\varepsilon_{EC}}-{\text{log}}_{2}\frac{1}{4\varepsilon_{PA}^{2}},$$ (12)

where $n_{x}$ is the number of total counts in the X basis and ${\bar{E}}_{p}$ is the upper bound of phase error rate under the X basis. $\lambda_{EC}=n_{x}\mathrm{fH}\left(E_{b}^{x}\right)$ is the leaked information during error correction, where f is the error correction efficiency, $E_{b}^{x}$ is the bit error rate under the X basis, and $H\left(x\right)=-x\;\log_{2}x-\left(1-x\right)\log_{2}\left(1-x\right)$ denotes the binary Shannon entropy. $\varepsilon_{EC}$ and $\varepsilon_{PA}$ are the failure probabilities for the error correction and privacy amplification, respectively, and we set $\varepsilon_{EC}=\varepsilon_{PA}=10^{-10}$.

$E_{b}^{x}$ can be obtained from experimental results and $E_{p}$ is bounded by the following inequality:

$$1-2\Delta \leq \sqrt{E_{b}^{y}E_{p}}+\sqrt{\left(1-E_{b}^{y}\right)\left(1-E_{p}\right)},$$ (13)

where $\Delta =\left(1-|\langle \Psi_{Y}{\left|\Psi_{X}\rangle \right|}^{2}\right)/2Q$. $Q=n_{\mathrm{tot}}/N$ is the total gain, where $n_{\mathrm{tot}}$ is the number of detection events and N is the number of pulses sent. $|\Psi_{X}\rangle$ $\left(|\Psi_{Y}\rangle \right)$ is the basis-dependent state under the X (Y) basis. The fidelity can be expressed as

$$\begin{array}{c} \begin{array}{c} \langle \Psi_{X}|\Psi_{Y}\rangle =\frac{1}{4}[\left(1-i\right)\langle \sqrt{\mu }|i\sqrt{\mu }\rangle +\left(1-i\right)\\ \;\langle -\sqrt{\mu }|-i\sqrt{\mu }\rangle +\left(1+i\right)\langle \sqrt{\mu }|-i\sqrt{\mu }\rangle \\ \;+\left(1+i\right)\langle -\sqrt{\mu }|i\sqrt{\mu }\rangle ]. \end{array} \end{array}$$ (14)

Considering the finite-key effect, Kato’s inequality [71] is utilized. The upper bound of the expectation value $m_{y}^{*}$ is given by $m_{y}+\Delta_{n_{y}}$, where $m_{y}=n_{y}E_{b}^{y}$ represents the number of errors in the Y basis and $\Delta_{n_{y}}=\sqrt{\frac{1}{2}n_{y}\ln\varepsilon_{F}^{-1}}$ with the failure probability $\varepsilon_{F}=10^{-10}$. ${E_{b}^{y}}^{*}=m_{y}^{*}/n_{y}$ can be calculated and $E_{p}^{*}$ can be derived according to Eq. (13). Thus, we can get the number of phase errors $m_{p}^{*}=n_{x}E_{p}^{*}$ and ${\bar{m}}_{p}$ can subsequently be estimated by the inequality. Consequently, we can get the upper bound of phase error rate ${\bar{E}}_{p}={\bar{m}}_{p}/n_{x}$. The security of 4-phase MDI-QKD is presented in Ref. [36].

### Two quantum datasets

In this subsection, we introduced the details of 2 quantum datasets used to evaluate the performance of QuNetQFL in entanglement classification and nonstabilizerness classification tasks. Entanglement is a fundamental resource for quantum information-processing tasks, especially in quantum communication. A given n-qubit state ψ is a product state if and only if $\psi ={⊗}_{i=1}^{n}\psi_{i}$; otherwise, it is entangled. For this task, we employed the NTangled dataset [72] which quantifies multipartite entanglement using Concentratable Entanglement (CE), defined as $C\left(\psi \right)=1-\frac{1}{2^{n}}\sum_{\alpha \in P}\mathrm{Tr}\left[\rho_{\alpha }^{2}\right]$. Here, P is the power set of the set $\left\{1,2,\ldots ,n\right\}$, and $\rho_{\alpha }$ is the reduced density operator with respect to the index α. States with higher $C\left(\psi \right)$ exhibit greater entanglement. Following the methods in Ref. [72], we generated a balanced 3-qubit dataset consisting of low (CE = 0.05) and high (CE = 0.35) entangled states using a 6-layer HEA. In an IID setting, each client accessed 160 states for training, while the server processed 200 test states with equal proportions from the 2 classes in both 3-client and 4-client scenarios.

Quantum magic, or nonstabilizerness [73,74], is crucial for achieving quantum computational advantage beyond classical simulation [75]. Stabilizer states, generated by Clifford operations, are classically simulable, whereas nonstabilizer states possess quantum magic. For this task, we employed a popular magic measure, *stabilizer Rényi entropy* [76], to quantity nonstabilizeness. A balanced 3-qubit dataset was generated, comprising nonstabilizer states with *stabilizer Rényi entropy* greater than 1.5 (sampled Haar-randomly) and stabilizer states selected from the total of 1,080 3-qubit stabilizer states. Each client accessed 120 training states, and the server processed 120 test states, with equal class proportions in both 3-client and 4-client scenarios.

For both classification tasks, we assume all clients are quantum enabled, equipped with quantum computing devices that can implement QNNs, while the server needs only to aggregate updates in classical parameter form and can therefore be entirely classical. However, the protocol is also compatible with a quantum server if needed to validate the performance of the aggregated quantum model at each step, potentially enabling early termination of the protocol when appropriate. We used QNNs with the HEA, as shown in Fig. 1C. To enhance the capacity of the learning model, we input the states parallelly twice and adopted a 6-qubit HEA with 4 layers. The label predictions were obtained by measuring the last qubit of the circuit on the Z basis. For the binary classification tasks considered here, a single readout qubit is sufficient to encode the prediction score after the variational circuit, while also reducing the measurement overhead on near-term quantum hardware. These 2 tasks were performed with 16-bit quantization (q=16) over 200 and 160 communication rounds, respectively. Each client conducted local training with a batch size of 32, an initial learning rate of 0.01 and 0.02 (for the 2 tasks, respectively), and the Adam optimizer, employing mean squared error as the loss function, with one local epoch per round.

### Federated fine-tuning a hybrid BERT–QNN model

In this subsection, we introduce the methods of federated fine-tuning a hybrid classical-quantum model, leveraging the power of pre-trained classical models for transfer learning. We use the LLM BERT as a pre-trained classical model to extract complex features, which are then processed on a quantum device through a QNN. When federated training such a model to solve real-world language-processing tasks, we freeze the parameters of BERT and fine-tuning only the parameters of the QNN and the connecting linear layer. This approach allows us to leverage the power of a pre-trained classical model while enhancing its performance with quantum processing. In the sentiment analysis task, the BERT–QNN architecture consists of BERT, followed by a linear layer that reduces the 768-dimensional BERT output to a 4-dimensional vector, and a 4-qubit, 3-layer QNN using a HEA. In this setup, only 3,112 parameters are fine-tuned, compared to the total over 100 million parameters of the BERT model. We note that our experimentally generated secret keys are sufficient to support this model size, with the number of communication rounds set to 100. For comparison, we also implemented a dimension-matched classical baseline in which the QNN module was replaced by a lightweight classical nonlinear classification head while keeping the frozen BERT encoder, the 4-dimensional bottleneck, the 4-client IID setting, 100 communication rounds, 16-bit quantization, and QKD-key-based secure aggregation unchanged. This baseline contains 3,081 trainable parameters. Details of the simulated setup are provided in the Supplementary Materials.

## Data Availability

All data that support the findings of this study are available from the corresponding authors upon reasonable request.
