# Supplementary material for: Experimentally Validated Quantum-Secure Federated Learning over a Multi-user Quantum Network
Source: Research (Wash D C). 2026 Jun 10;9:1299. doi: 10.34133/research.1299 (PMC13250279; doi:10.34133/research.1299)
Supplement: Supplementary 1 — Fig. S1 Tables S1 to S4 [file research.1299.f1.zip › Table S4 Primers.docx]

**Table S4 Primers used in the present study**

| **Gene** | **Primer sequence** |
| --- | --- |
| **qRT-PCR** |  |
| METTL3 | forward: 5’- CTATCTCCTGGCACTCGCAAGA -3’ |
|  | reverse: 5’- GCTTGAACCGTGCAACCACATC-3’ |
| MSH5 | forward: 5’- CCGATTCCACTGCTGATCCC-3’ |
|  | reverse: 5’- ATGAGTCGGACGTGTGAACC -3’ |
| IFNβ | forward: 5’- TGGCTGGAATGAGACTATTGTT -3’ |
|  | reverse: 5’- GGTAATGCAGAATCCTCCCATA-3’ |
| IL6 | forward: 5’- CACTGGTCTTTTGGAGTTTGAG -3’ |
|  | reverse: 5’- GGACTTTTGTACTCATCTGCAC-3’ |
| GAPDH | forward: 5’- GCACCGTCAAGGCTGAGAAC-3’ |
|  | reverse: 5’- TGGTGAAGACGCCAGTGGA -3’ |
| CXCL10 | forward: 5’- CTCTCTCTAGAACTGTACGCTG-3’ |
|  | reverse: 5’- ATTCAGACATCTCTTCTCACCC-3’ |
| Pre-cGAS-1 | forward: 5’- CAGACTGAAGTGCGACTCCGCGTTC -3’ |
|  | reverse: 5’- GACCCGGGGAAGGTAGGGACTGC -3’ |
| Pre-cGAS-2 | forward: 5’- GGAAGAAATTAACGACATTAAAGG-3’ |
|  | reverse: 5’- AATGGGAGTGTACATTGGCAAT-3’ |
| cGAS | forward: 5’- GCTGCTCCACGAAGCCAAGAC-3’ |
|  | reverse: 5’- GCGGCTGAGCTTCAACTTCTCC-3’ |
| STING | forward: 5’- GCCCTGTTGCTGCTGTCCATC-3’ |
|  | reverse: 5’- GGATGTTCAGTGCCTGCGAGAG-3’ |
| IRF3 | forward: 5’- GCAGGAGGATTTCGGAATCTTC-3’ |
|  | reverse: 5’- GGAAATTCCTCTTCCAGGTTGG-3’ |
| YTHDF2 | forward: 5’- AGCCTCTTGGAGCAGTACAAA-3’ |
|  | reverse: 5’- GCATTATTGGGCCTTGCCTG-3’ |
| METTL3 | forward: 5’- CTATCTCCTGGCACTCGCAAGA -3’ |
|  | reverse: 5’- GCTTGAACCGTGCAACCACATC-3’ |
| ALKBH5 | forward: 5’- CCAGCTATGCTTCAGATCGCCT -3’ |
|  | reverse: 5’- GGTTCTCTTCCTTGTCCATCTCC-3’ |
| HPRT | forward: 5’- TGACACTGGCAAAACAATGCA-3’ |
|  | reverse: 5’- GGTCCTTTTCACCAGCAAGCT-3’ |
| 18S rRNA | forward: 5’- CGGACAGGATTGACAGATTGATAGC-3’ |
|  | reverse: 5’- TGCCAGAGTCTCGTTCGTTATCG -3’ |
| Firefy-Luc | forward: 5’- GGTACTGTTGGTAAAGCCAC-3’ |
|  | reverse: 5’- CTCTTCATAGCCTTATGCAG-3’ |
| Renilla-Luc | forward: 5’- CAATGGGCAGGTGTCCACTC -3’ |
|  | reverse: 5’- GTTCTGGATCATAAACTTTC -3’ |
| **Mutation plasmid** | **Primer sequence** |
| MSH5/CDS-Mut | forward: 5’- agatcctaac ctggGcttga acgttttcat -3’ |
|  | reverse: 5’- atgaaaacgt tcaagCccag gttaggatct-3’ |
| cGAS/CDS-Mut | forward: 5’- tttcttcagtgcctcaggCcagaaaaacttgagaatt-3’ |
|  | reverse: 5’- aattctcaagtttttctgGcctgaggcactgaagaaa-3’ |
| **SELECT** | **Primer sequence** |
| MSH5-X | forward: 5’-tagccagtaccgtagtgcgtgggctcatgaaaacgttcaag-3’ |
|  | reverse: 5phos/ccaggttaggatcttccaaacagaggctgagtcgctgcat -3’ |
| RRM2B-N | forward: 5’-tagccagtaccgtagtgcgtgttcctggctcatgaaaacgt-3’ |
|  | reverse: 5’- 5phos/caagtccaggttaggatcttcagaggctgagtcgctgcat-3’ |
| cGAS-X | forward: 5’- tagccagtacgtagtgcgtgtctcaagtttttctg -3’ |
|  | reverse: 5phos/cctgaggcactgaacagaggctgagtcgctgcat -3’ |
| cGAS-N | forward: 5’- tagccagtacgtagtgcgtgcaagtttttctgtcc-3’ |
|  | reverse: 5’- 5phos/gaggcactgaagaaacagaggctgagtcgctgcat-3’ |
